# Surgical re-evaluation patterns among pediatric vagus nerve stimulation non-responders: a single-center retrospective study

**DOI:** 10.1007/s00381-026-07299-5

**Published:** 2026-05-05

**Authors:** James M. Mossner, Sunny Abdelmageed, Ryan Wang, Kristina Terrani, Megan Votoupal, Klaudia Dziugan, Heba Akbari, Rachel Pauley, David Bieber, Sandi K. Lam, Jeffrey S. Raskin

**Affiliations:** 1 Department of Neurosurgery, McGaw Medical Center of Northwestern University, Northwestern University Feinberg School of Medicine, 251 E Huron St, Chicago, IL 60611 USA; 2 Division of Pediatric Neurosurgery, Ann & Robert H. Lurie Children’s Hospital, Chicago, IL USA; 3 Department of Neurosurgery, George Washington University, Washington, DC USA; 4 Department of Neurology, Ann & Robert H. Lurie Children’s Hospital of Chicago, Chicago, IL USA; 5 Department of Pediatrics, Northwestern University Feinberg School of Medicine, Chicago, IL USA

**Keywords:** Vagus nerve stimulation, Epilepsy, Drug-resistant, Pediatric, Adolescent

## Abstract

**Objective:**

Vagus nerve stimulation (VNS) is an established adjunctive therapy for pediatric drug-resistant epilepsy (DRE). While ~ 50% of patients achieve clinically meaningful seizure reduction, non-responders may not undergo further evaluation for additional surgical options, leaving persistent risk for uncontrolled seizures and epilepsy-related morbidity and mortality. We evaluated rates of post-VNS surgical re-evaluation and epilepsy surgery among pediatric VNS non-responders and secondarily assessed seizure and antiseizure medication (ASM) outcomes.

**Methods:**

We performed a retrospective review of pediatric patients with DRE who underwent VNS implantation at our institution (2013–2020). Patients were classified as “responders” if they achieved ≥ 50% seizure frequency reduction from baseline. Variables included demographics, operating surgeon, generator model, revision history, seizure type and frequency, and ASM burden. Groups were compared using chi-square and Student’s *T*-tests.

**Results:**

Forty-seven patients (25 responders, 22 non-responders) met inclusion criteria. Groups did not differ significantly in age, sex, race, operating surgeon, generator model, revision history, or baseline ASM burden. At follow-up, responders required fewer ASMs than non-responders (*p* = 0.03). Among non-responders, only 3/22 patients (13.6%) underwent additional epilepsy surgery (two responsive neurostimulation, one corpus callosotomy), and all achieved meaningful seizure reduction postoperatively. Three deaths occurred in the non-responder group, none surgery-related.

**Conclusion:**

Nearly half of pediatric patients treated with VNS failed to achieve seizure reduction, yet few underwent re-evaluation for further surgical management despite favorable outcomes among those who did. Structured follow-up pathways are needed to ensure timely surgical re-assessment of pediatric VNS non-responders within comprehensive epilepsy programs.

## Introduction

Drug-resistant epilepsy (DRE) is defined by the persistence of seizures despite two or more appropriately chosen and adequately trialed antiseizure medications [1]. The prevalence of DRE in community-based pediatric populations is approximately 13.7% [2]. Children with DRE should be referred to a comprehensive epilepsy center for assessment of surgical candidacy. However, not all patients are suitable for resective, ablative, or disconnection procedures. Neuromodulation represents an effective treatment modality for patients with nonlesional, multifocal, or bilateral seizure onset or for those with seizure foci in eloquent cortex [3, 4].

VNS is approved by the Food and Drug Administration (FDA) for children (ages 4 to 18). The device consists of helical coil electrodes wrapped around the left vagus nerve and connected to an internal pulse generator (IPG) positioned in the chest wall. VNS is typically offered to patients with multifocal and/or non-localizable refractory epilepsy who are not candidates for resective surgery [5]. “Responders” are generally defined as patients who achieve a ≥ 50% reduction in seizure frequency, with approximately 57% meeting this criterion after an average follow-up of 2.5 years [6]. Complete seizure freedom is achieved in up to 12% of patients in individual studies [7].

DRE carries substantial morbidity, including increased risk of physical injury, cognitive decline, and mortality, as well as a profound reduction in quality of life [8]. Long-term outcomes in pediatric DRE vary significantly depending on the treatment modality employed. In a cohort of 18,292 children, 10-year survival rates were highest among those who underwent cranial epilepsy surgery (98.45%), followed by VNS-treated patients (92.65%), and lowest among those managed medically (89.27%). Compared with the medically managed cohort, the hazard ratio for all-cause mortality was significantly lower among patients treated with VNS (0.60) and those who underwent cranial surgery (0.19) [9].

Despite these encouraging outcomes, only one- to two-thirds of patients achieve a meaningful response, leaving a substantial proportion with persistent, uncontrolled seizures. These non-responders remain vulnerable to progressive neurological decline, physical injury, diminished quality of life, and elevated mortality relative to age-matched peers [10–13]. In clinical practice, children who do not respond to VNS are often regarded as having exhausted surgical options, and many are not re-evaluated for advanced interventions such as disconnection or responsive neurostimulation surgery. There is limited literature describing the timing, frequency, and outcomes of repeat Phase I evaluations or subsequent epilepsy surgeries offered to pediatric VNS non-responders.

This study primarily aimed to quantify post-VNS surgical re-evaluation and subsequent epilepsy surgery among pediatric VNS non-responders at our institution. Secondary aims were to compare seizure outcomes (total weekly seizure frequency; Engel class) and ASM burden between responders and non-responders.

## Methods

### Patient selection

**Fig. 1:**
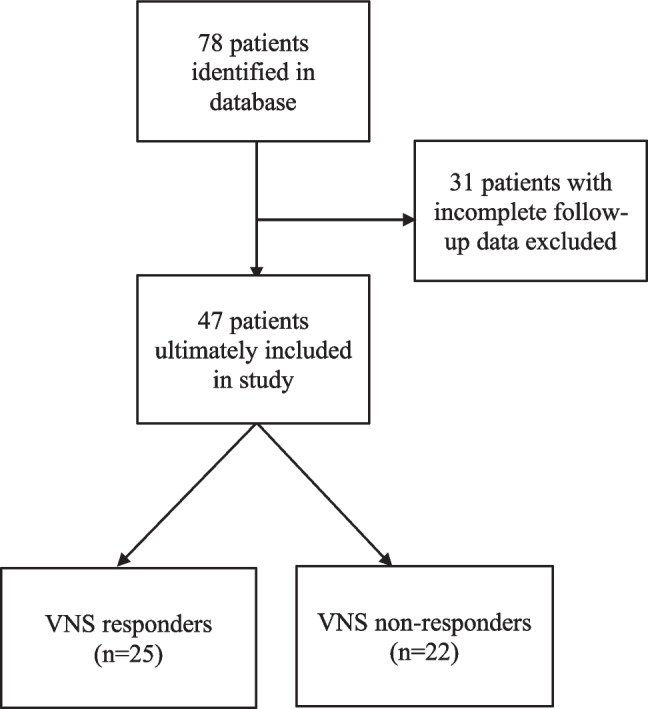
Patient selection. Of 78 pediatric patients identified who underwent VNS implantation, 31 were excluded due to incomplete follow-up. The final cohort included 47 patients, with 25 classified as responders and 22 as non-responders based on reported seizure reduction

This study was approved by the Ann & Robert H. Lurie Children’s Hospital of Chicago Institutional Review Board (IRB# 2024-6558). A retrospective chart review was conducted of pediatric patients who underwent first-time VNS placement for DRE from January 1, 2013, to December 31, 2020. A minimum follow-up of 12 months after implantation was required for inclusion*.* Patients lacking the minimum follow-up or adequate seizure outcome documentation were excluded. Patient identification, exclusions, and the final analytic cohort are summarized in Fig. 1.

### Patient factors

Demographic factors included date of age at implantation, sex, and self-reported race or ethnicity. Clinical variables included baseline seizure frequency, number and type of antiseizure medications (ASMs), VNS generator model, implanting surgeon, and any subsequent epilepsy procedures, including VNS revision procedures. In this cohort, all subsequent revision surgeries were elective VNS battery replacements; there were no unplanned VNS lead revisions. Assessments were obtained from documentation at the preoperative baseline and the most recent postoperative follow-up. Subsequent epilepsy procedures were recorded, including phase two intracranial monitoring, resective surgery, responsive neurostimulation (RNS), deep brain stimulation (DBS), corpus callosotomy, and any VNS revisions. Surgery location was categorized as procedures performed at Lurie Children’s Hospital versus external institutions.

### Outcome measures and data analysis

The primary outcome was post-VNS surgical re-evaluation and subsequent epilepsy surgery among VNS non-responders (e.g., intracranial monitoring, resective/disconnective surgery, RNS, and DBS). Secondary outcomes included total weekly seizure frequency, Engel class at most-recent follow-up, and ASM burden. Seizure type and frequency were determined from clinical documentation during visits with the pediatric neurosurgery division or the comprehensive epilepsy center. Reported seizure count ranges were averaged to yield an estimated weekly frequency. Seizure outcomes were categorized using the Engel classification scale at each follow-up interval, and responder status was assessed based on this synthesized information. Responders were defined as patients who achieved a ≥ 50% reduction in seizure *frequency* relative to baseline. Engel classification outcomes were reported as Classes I–IV. Data from each patient’s most recent follow-up visit were used to evaluate long-term seizure outcomes.

Paired Student’s *T*-tests (two-tailed, *p* ≤ 0.05 considered significant) were used to compare seizure frequency at each follow-up interval with baseline values. Responders and non-responders were separated into two distinct groups and Student’s *T*-tests were employed to examine differences in relevant demographic and clinical factors. All statistical analyses were completed using RStudio (RStudio Team, 2023).

## Results

### Cohort demographics

Forty-seven pediatric patients underwent first-time VNS implantation at a single tertiary epilepsy center during the study period. The average age at implantation was 10.9 years (range 2.3–21.4 years; IQR 8.3 years). The most common epilepsy etiologies were generalized tonic, generalized clonic, and myoclonic seizure types, among others. Patients achieving ≥ 50% reduction in postoperative seizure frequency compared with baseline were classified as responders; all others were considered non-responders according to this study’s definition. There were no significant differences between responders and non-responders in age at surgery (*p* = 0.51), sex (*p* = 0.71), race (*p* = 0.11), surgeon (*p* = 0.75), generator model (*p* = 0.67), or revision history (*p* = 0.36; Table 1). Ethnicity was statistically associated with treatment response in this cohort (*p* = 0.003), with Hispanic patients less likely to meet responder criteria compared to non-Hispanic patients; however, given the small sample size and retrospective design, this observation should be interpreted cautiously and warrants validation in larger, multi-institutional studies.

**Table 1:** Cohort demographics

	Responder (R, *n* = 25)	Non-responder (NR, *n* = 22)	*P*-value R-NR
Mean age at surgery (Yrs)	14.0 ± 19.4	11.0 ± 5.58	0.51
Sex			0.71
Male (*n*)	15	12	
Female (*n*)	10	10	
Race			0.11
White (*n*)	14	13	
African American (*n*)	2	2	
Asian (*n*)	1	5	
Other (*n*)	8	2	
Ethnicity			**0.003**
Non-Hispanic (*n*)	23	12	
Hispanic (*n*)	2	10	
Surgeon			0.75
A (*n*)	3	2	
B (*n*)	17	16	
C (*n*)	3	4	
Other (*n*)	3	0	
Original generator model			0.67
Model 103 (*n*)	5	6	
Model 106 (Aspire, *n*)	3	1	
Model 1000 (Sentiva, *n*)	16	13	
Model 102 (*n*)	0	0	
Model 106 (*n*)	1	2	
Model 105 (*n*)	0	0	
Revision history*			0.36
No revision (*n*)	15	16	
Revision (*n*)	10	6	
Engel classification			
Engel I (*n*)	0	0	
Engel II (*n*)	2	0	
Engel III (*n*)	20	5	
Engel IV (*n*)	3	17	
Mortality (*n*)	0	3	--

*Subsequent revision surgeries were elective VNS battery replacements; there were no unplanned VNS lead revisions

### Clinical course following initial VNS implantation

Among 22 non-responders (46.8% of the cohort), only 3 (13.6%) underwent additional epilepsy surgery—two received RNS implants and one underwent corpus callosotomy. The reasons other non-responders did not proceed to additional epilepsy surgery (e.g., lack of referral for repeat evaluation, determination of non-candidacy, or family preference) were not consistently documented and could not be reliably adjudicated. All three patients achieved meaningful seizure reduction following their subsequent surgical intervention. No patients in the VNS responder group required additional epilepsy surgery in our study. During follow-up, three deaths occurred among non-responders, whereas none occurred in responders; no mortality was procedure-related.

### Number of ASMs taken at baseline and follow-up between VNS responders and non-responders

Baseline ASM burden did not differ significantly between responder and non-responder groups (*p* = 0.84).

At the most recent follow-up, responders required significantly fewer ASMs than non-responders (*p* = 0.03; Table 2). Non-responders also required more ASMs at follow-up compared with their own preoperative baseline (*p* = 0.02; Table 2).

**Table 2:** Number of ASMs taken between VNS responders and non-responders

	Responder (R)	Non-responder (NR)	*P*-value R-NR
Number of ASMs (*n*)			0.21
Preoperatively	3.17 ± 0.92	3.10 ± 1.21	0.84
Most-recent follow-up	2.84 ± 0.85	3.55 ± 1.26	**0.03**
* P*-value preop-follow-up	0.23	**0.02**	

### Total seizure frequency

Using total weekly seizure frequency as documented in clinical follow-up, responders demonstrated a significant reduction at the most recent follow-up compared with baseline (*p* = 0.02; Table 3). Non-responders did not demonstrate a significant change from baseline (*p* = 0.33; Table 3). There were no statistically significant between-group differences in total weekly seizure frequency at the most recent follow-up (*p* = 0.09; Table 3).

**Table 3:** Seizure type frequency between VNS responders and non-responders

	Responder (R)	Non-responder (NR)	*P*-value R vs NR
Seizure type I frequency			
Baseline (seizures/week)	37.3 ± 75.6	20.5 ± 30.6	0.31
Most-recent follow-up (seizures/week)	11.1 ± 20.3	40.4 ± 88.6	0.14
*P*-value baseline-follow-up	**0.04**	0.28	
Seizure type II frequency			
Baseline (seizures/week)	34.5 ± 58.6	19.1 ± 31.0	0.41
Most-recent follow-up (seizures/week)	5.05 ± 7.56	22.1 ± 32.7	0.22
*P*-value baseline-follow-up	0.20	0.14	
Total postoperative seizure			
Baseline (seizures/week)	59.8 ± 113.6	28.9 ± 43.0	0.21
Most-recent follow-up (seizures/week)	12.5 ± 20.2	47.6 ± 90.3	0.09
*P*-value baseline-follow-up	**0.02**	0.33	

## Discussion

DRE remains a major cause of morbidity and mortality in the pediatric population. VNS therapy was performed in 47 patients at our comprehensive pediatric epilepsy center during the study period as a treatment for DRE. Consistent with prior reports, approximately one- to two-thirds of patients with VNS fail to achieve a clinically meaningful reduction in seizure burden [10, 14]. Our data demonstrate that non-responders are infrequently re-evaluated for candidacy for additional surgical interventions, despite ongoing medically refractory seizures. Numerous studies have examined predictors of VNS response, yet reported findings remain inconsistent [15–24]. A recent meta-analysis identified shorter epilepsy duration before VNS implantation as a significant predictor of treatment success [22]. Other investigations have evaluated biomarkers such as heart rate variability and interictal EEG characteristics as potential predictors of VNS outcomes [16, 18, 20].

Connectomic studies have shown that pediatric VNS responders exhibit stronger left-hemispheric functional network connectivity, suggesting a potential avenue for response prediction [17]. Despite these promising lines of investigation, no reliable clinical, structural, or biological markers currently exist to predict VNS responsiveness. In contrast, there is limited research describing the clinical course of VNS non-responders and whether they subsequently undergo further surgical evaluation. We therefore examined the characteristics of VNS responders and non-responders at our institution, with a particular focus on surgical follow-up and re-evaluation patterns.

In our cohort, Hispanic ethnicity was statistically associated with lower likelihood of VNS response (*p* = 0.003). Some studies have similarly reported that Hispanic children with epilepsy experience longer time to seizure remission and lower overall rates of seizure control compared to non-Hispanic white peers, even after adjusting for socioeconomic and demographic variables [25, 26]. Subsequent analyses of prescription refill patterns and medication adherence in Hispanic pediatric patients did not explain these disparities in seizure control [27]. The underlying association remains unclear and may reflect unmeasured social, cultural, or biological factors rather than treatment resistance. Hispanic children have also been shown to be underrepresented among epilepsy surgical patients, further limiting population-level understanding of treatment outcomes [28–30]. While this finding reached statistical significance, it should be interpreted cautiously given the small sample size and single-center design. More importantly, the broader issue revealed by our data is that very few non-responders in this single-institution series, regardless of demographic background, undergo repeat surgical evaluation.

Consistent with prior studies, VNS responders required fewer ASMs at follow-up, whereas non-responders did not experience a reduction [31, 32]. Non-responders demonstrated an increase in ASM burden over time, reflecting ongoing medical intractability. Three deaths occurred among non-responders, compared with none among responders. These findings underscore the elevated morbidity and mortality risk faced by children with persistent DRE despite VNS therapy.

Although VNS was the first neuromodulatory device approved for DRE, there are now three available neuromodulatory options. An updated treatment algorithm proposed by Benbadis et al. recommends consideration of deep brain stimulation (DBS) following inadequate response to VNS [33]. In a partially randomized pediatric trial, the addition of DBS for VNS non-responders achieved a 39.6% greater seizure reduction compared with continued VNS alone [34]. There is also evidence supporting the combined or sequential use of VNS and RNS in children. Khankhanian et al. reported two pediatric patients who underwent RNS implantation after VNS, both of whom experienced improvement in seizure reduction [35]. Notably, both patients ended up with an unrelated disruption in their VNS systems which was causative of worsened seizure control. One patient exhibited worsening of his right-sided seizures only after VNS battery depletion which improved following replacement. Another study involving 64 patients who underwent concurrent VNS and RNS placement found a 64% responder rate after an average follow-up of 28 months [36]. These findings suggest complementary and potentially synergistic neuromodulatory effects when combining intra- and extracranial neurostimulation.

Although limited, the existing evidence supports the potential benefit of concurrent or add-on neuromodulatory approaches following VNS non-response. In our cohort, all three non-responders who underwent subsequent surgery achieved meaningful seizure reduction, underscoring the potential benefit of revisiting surgical options after VNS non-response. These findings are consistent with prior studies demonstrating improved seizure control among pediatric VNS non-responders who later undergo disconnective or neuromodulatory procedures. For example, a recent multicenter series evaluating corpus callostomy in pediatric VNS non-responders with Lennox-Gastaut Syndrome reported a 60–83% postoperative reduction in drop attacks relative to baseline [37]. Similarly, the prospective randomized patient preference trial (ADVANCE trial) found that adding anterior nucleus DBS for patients with inadequate VNS response led to a 52% seizure reduction, compared with 12% among those who continued VNS alone [34].

Given the persistent, worsening seizure burden, elevated mortality risk, and accumulating evidence that additional neuromodulatory or disconnective procedures can improve seizure outcomes, it is imperative that pediatric VNS non-responders should be systematically re-entered into the surgical epilepsy evaluation pathway to be considered for additional intervention. Establishing structured follow-up protocols within comprehensive epilepsy programs may help ensure timely identification of candidates for reoperation and ultimately improve long-term outcomes.

Factors of multidisciplinary epilepsy care and VNS management are essential: the largest single-institution retrospective series of 400 pediatric VNS patients treated accordingly to an institutional protocol demonstrated a 90.5% responder rate at last follow-up, with 20.5% of patients with seizure freedom. In that study, duration of epilepsy and early VNS implantation within 2 years of seizure onset was associated with more favorable outcomes. Higher stimulation parameters according to their standardized treatment protocol was also a variable correlating with more favorable seizure outcomes [38]. Our cohort does not compare favorably to these outcomes, which highlights an immediate direction for future research and action.

## Limitations

This study is a single-center retrospective chart review, which may limit generalizability and introduce selection bias. We could not reliably determine why most VNS non-responders did not undergo repeat surgical evaluation or subsequent surgery (e.g., referral patterns, candidacy determinations, patient/family preferences, or follow-up outside our system), which limits causal interpretation of the observed care gap. There was no multidisciplinary treatment protocol or pathway in place for the duration of the study. VNS programming parameters are not known. Though the number of antiseizure medications pre- and post-VNS is recorded, the duration of epilepsy prior to VNS implantation is not known. The diagnosis and etiology of seizures are not included. Labeling of seizure types in the electronic medical records was consistent for each individual patient but not across patients; thus, the specific types of seizures are not specified in this study. Seizure frequency was determined from family-reported counts during outpatient follow-up visits rather than from objective seizure diaries or electronic tracking devices. This reliance on subjective reporting introduces potential recall bias and variability in outcome assessment. Additionally, systemic differences in how seizure activity was reported, observed, or documented may have influenced the classification of responders versus non-responders. Provider expectations, documentation practices, or implicit biases may also have contributed to differences in recorded seizure outcomes. Accordingly, we emphasize total seizure frequency rather than seizure-subtype analyses. While Hispanic ethnicity was analyzed as a variable in this study, primary language and use of interpreter services were not captured. Language barriers and cultural factors may have added complexity to provider-family communication, potentially affecting the accuracy and interpretation of seizure reporting.

## Conclusion

Nearly half of pediatric patients treated with VNS in this cohort did not achieve a meaningful reduction in seizure frequency. Non-responders demonstrated persistently high seizure burden and experienced greater morbidity and mortality compared with responders. Despite this elevated risk, only three of twenty-two non-responders (13.6%) underwent additional epilepsy surgery, and all achieved meaningful seizure reduction following re-intervention. These findings highlight a critical gap in care: VNS therapy should not be a terminal step in epilepsy treatment pathways. Our study findings emphasize the opportunity for treatment pathways highlighting the importance of systematically re-entering VNS non-responders into the Phase I work-up of the surgical evaluation pathway to reconsider advanced treatment options, including disconnective or neuromodulatory procedures.

## Data Availability

De-identified data are available from the corresponding author upon reasonable request, subject to institutional approvals.
